# Ocean Warming May Enhance Biochemical Alterations Induced by an Invasive Seaweed Exudate in the Mussel *Mytilus galloprovincialis*

**DOI:** 10.3390/toxics9060121

**Published:** 2021-05-28

**Authors:** Hugo C. Vieira, Andreia C. M. Rodrigues, Sílvia F. S. Pires, Jacinta M. M. Oliveira, Rui J. M. Rocha, Amadeu M. V. M. Soares, Maria D. Bordalo

**Affiliations:** CESAM—Centre for Environmental and Marine Studies, Department of Biology, University of Aveiro, 3810-193 Aveiro, Portugal; rodrigues.a@ua.pt (A.C.M.R.); silviapires1@ua.pt (S.F.S.P.); jacintaoliveira@ua.pt (J.M.M.O.); ruimirandarocha@ua.pt (R.J.M.R.); asoares@ua.pt (A.M.V.M.S.); maria.bordalo@ua.pt (M.D.B.)

**Keywords:** invasive macroalgae, marine species, biochemical markers, warming scenario

## Abstract

Ocean warming and biological invasions are among the most pervasive factors threatening coastal ecosystems with a potential to interact. Ongoing temperature rise may affect physiological and cellular mechanisms in marine organisms. Moreover, non-indigenous species spread has been a major challenge to biodiversity and ecosystem functions and services. The invasive red seaweed *Asparagopsis armata* has become successfully established in Europe. Its exudate has been considered deleterious to surrounding native species, but no information exists on its effect under forecasted temperature increase. This study evaluated the combined effects of temperature rise and *A. armata* exudate exposure on the native mussel *Mytilus galloprovincialis*. Oxidative stress, neurophysiological and metabolism related biomarkers were evaluated after a 96 h-exposure to exudate (0% and 2%) under present (20 °C) and warming (24 °C) temperature scenarios. Short-term exposure to *A. armata* exudate affected the oxidative stress status and neurophysiology of the mussels, with a tendency to an increasing toxic action under warming. Significant oxidative damage at protein level was observed in the digestive gland and muscle of individuals exposed simultaneously to the exudate and temperature rise. Thus, under a climate change scenario, it may be expected that prolonged exposure to the combined action of both stressors may compromise *M. galloprovincialis* fitness and survival.

## 1. Introduction

Global warming as a consequence of ongoing greenhouse gas emissions (e.g., atmospheric CO_2_) derived from anthropogenic activities has been a topic of increasing concern and represent a major threat to the marine environment [1]. As a result, since 2005 the ocean has been getting progressively warmer, and it is expected that by 2100 ocean temperature will increase by two to four times for low emissions (RCP2.6) and five to seven times for the high emission scenario (RCP8.5) compared to the observed changes since 1970 [1]. Warming ocean is affecting marine life at all levels of biological organization, which impacts not only marine ecosystems but also human communities depending on them [1]. As ectotherms, the majority of aquatic species may be particularly affected by increasing temperature since they are sensitive to thermal stress [2]. Consequently, their physiological and metabolic processes may be altered as compensatory mechanisms once their optimal thermal tolerance range is exceeded [3]. For instance, oxidative stress is expected as temperature rise can enhance the formation and release of reactive oxygen species (ROS) above the normal background levels, contributing to cell and tissue damage, effect on the organism being dependent on their ability to activate antioxidant defense mechanisms [4].

Beside warming, biological invasions are expected to increase at accelerating rates in the next decades as a result of globalization and are a growing ecological and socioeconomic concern, due to the profound impacts they may pose in invaded ecosystems [5]. Non-native species that successfully establish may disrupt resident communities, and are a major cause for biodiversity loss and ecosystem functioning impairment [6]. Invasive seaweeds in particular have caused significant changes to the composition of coastal communities by displacing native flora and interfering with trophic food chains [7]. The red seaweed *Asparagopsis armata* Harvey 1855 (native from the Southern Australia and New Zealand) is no exception and has been found successfully widespread across Mediterranean and Atlantic coasts [8,9] owing to its strong invasive behavior. Both the gametophyte phase of *A. armata,* with its typical harpoon-like branches that hook on other marine organisms and floating material, and the free-floating tetrasporophyte phase contribute to *A. armata* invasion success [10,11]. This species has the capacity to rapidly spread, being able to colonize invaded areas, inflicting changes in the associated communities. These alterations may be observed not only in terms of macroinvertebrates [9] but also of other seaweeds [12], possibly due to competition for resources (space and nutrients) and to the exudation of chemical compounds into the tide pools [13,14] produced as a chemical defense [10,15]. In fact, this red macroalga has been found to pose toxic effects to the physiological status of surrounding biota [16,17] when in contact with its exudate and even deterred feeding from potential consumers [18,19]. Algae from the genus *Asparagopsis* are a great source of halogenated organic compounds (such as halomethanes, haloacetates, haloacetones) and other secondary metabolites [14], which are thought to be the major responsible for these effects. In fact, similarly to the temperature rise, halocarbons are also potent ROS generators [20].

The Mediterranean mussel *Mytilus galloprovincialis* (Lamarck, 1989) presents a worldwide distribution and a sessile behavior, with tolerance to a variety of stressors and a capacity to accumulate pollutants reflecting their presence in the environment, being widely used as a sentinel and a bioindicator species in coastal environments [21,22,23]. Furthermore, this mussel is not only an ecologically important species, but also has a high commercial value [24,25,26]. Thus, any impact on its populations may have implications for community structure and functioning of coastal ecosystems, as well as for human consumers. *M. galloprovincialis* was recently described to be affected by the exudate released by *A. armata* in terms of energy consumption, clearance capacity and attachment strength [16]. Although increased heat is known to inflict physiological and biochemical stress in this mussel species [26,27,28], there is no knowledge on the combined effects of *A. armata* exudate and temperature rise. However, it is widely accepted that an increase in the water temperature may modify the ability of the organisms to cope with additional stressors [29,30,31,32], so we hypothesize that warming seawater may potentiate the toxic effects of *A. armata* exudate in *M. galloprovincialis*.

In this sense, the present study aimed to evaluate if a short-term experimental warming alters the effect of *A. armata* exudate on the biochemical response of the mussel *M. galloprovincialis*. Energy reserves, electron transport system (ETS) and lactate dehydrogenase (LDH) were evaluated as biomarkers of energy alteration and metabolism, catalase (CAT), glutathione-s-transferase (GST) and heat shock proteins (HSP) as biomarkers of oxidative stress, lipid peroxidation (LPO) and protein carbonyl (PC) levels as biomarkers of oxidative damage, acetylcholinesterase (AChE) activity as a neurotoxicity endpoint.

## 2. Materials and Methods

### 2.1. Asparagopsis Armata Sampling and Exudate Production 

The gametophyte phase of the red macroalga *A. armata* was collected by hand through free diving in a subtidal zone at the Terceira island Azores (Portugal) (38°38′59.2″ N, 27°13′16.4″ W). The macroalgae were kept in aerated seawater tanks until next day and packed in sealed containers to be transported to the laboratory in Aveiro (Portugal). Immediately upon arrival, *A. armata* was cleared from visibly associated fauna and debris. They were then allocated to a tank with artificial seawater (marine RedSea^®^ Salt premium grade) in a 1:10 proportion (salinity 35 ± 1, pH 8.0 ± 0.1, temperature 20.0 ± 0.5 °C) in the dark and no aeration for 24 h to produce the exudate, as in [16]. Afterwards, the alga was removed from the tank and the resulting media (considered as the stock solution and representing 100% of exudate) was preserved at –20 °C, until further use.

### 2.2. Mytilus galloprovincialis Sampling and Acclimatization

*Mytilus galloprovincialis* mussels (4 ± 0.5 cm length) were collected in July 2020 at the intertidal rocky shores of Barra de Aveiro, at the northwest of Portugal (40°38′38.8″ N, 8°44′44.6″ W) during low tide. Organisms were immediately transported to the laboratory and cleaned of superficial debris and encrusting organisms. Afterwards, they were acclimated to controlled laboratory conditions for seven days in aquaria containing aerated artificial seawater (salinity 35 ± 0.5, pH 8.0 ± 0.1, oxygen saturation > 80%) in a recirculating aquatic system (flow-through system ensured continuous seawater renewal), without being fed. Half of the mussels were maintained at a temperature 20.0 ± 0.5 °C and the remaining mussels were acclimated to a temperature of 24.0 ± 0.5 °C. All individuals were maintained under a 14 h light: 10 h dark photoperiod.

### 2.3. Exposure Assay

Twenty-eight *M. galloprovincialis* were randomly distributed into individual 1L aquaria containing 500 mL of experimental media (1 individual per aquarium). Mussels were exposed for 96 h to the following experimental conditions replicated 7 times: 0% exudate at 20 °C, 2% exudate at 20 °C, 0% exudate at 24 °C and 2% exudate at 24 °C. Temperature increase of 4 °C was based on the temperature scenarios projected for 2100 [1]. Temperature was maintained constant by keeping the aquaria in a water bath, adapted from [33]. Water bath temperature was controlled through water chillers (Hailea, HC-300A, Chaozhou, China) and submergible water heaters (Prodac Magictherm 150w, Cittadella, Italy). The 2% exudate concentration was chosen according to [16] sublethal toxicity test results. During exposure, physical-chemical parameters were maintained as in the acclimation period. After 96h, *M. galloprovincialis* individuals were dissected, their tissues (gills, digestive gland, muscle) were separated, weighed and immediately frozen in liquid nitrogen. Samples were stored at –80 °C until further biochemical analysis.

### 2.4. Biomarker Analysis

#### 2.4.1. Sample Preparation for Biomarkers Analysis

Each sample of mussel tissue was homogenized on ice by sonication (30 s 10% pulsed mode, 250 Sonifier, Branson Ultrasonics) using ultra-pure water (1800 µl per gills sample, 117.4 ± 33.6 mg mean weight; per digestive tissue sample, 140.3 ± 39.8 mg mean weight; per muscle sample, 167.7 ± 55.9 mg mean weight). 

Gill and digestive tissue sample homogenates were diluted 1:1 with 0.2 M K-phosphate buffer, pH 7.4 (centrifuged for 15 min at 10,000 g 4 °C). The obtained post-mitochondrial supernatant (PMS) was split into microtubes and kept in –80 °C for posterior analyses of oxidative stress-related biomarkers. Lipid peroxidation (LPO) was evaluated in an aliquot containing 4% butylated hydroxytoluene (BHT) in methanol. Two aliquots were stored for heat shock proteins (HSP) and protein carbonylation (PC) determination. 

Muscle sample homogenates were split into 3 aliquots to analyze lipid, sugar and protein contents, and electron transport system (ETS) activity. One aliquot for LPO determination was also stored as previously described. Aliquots for lactate dehydrogenase (LDH) and protein carbonylation (PC) determination were also stored. 

Micro-assays were set up in 96 well flat bottom plates and all biomarkers determinations were read spectrophotometrically (Microplate reader MultiSkan Spectrum, Thermo Fisher Scientific, Waltham, MA, USA).

#### 2.4.2. Oxidative Stress-Related Biomarkers

According to the Bradford method [34], the protein concentration of PMS was determined using bovine γ-globulin as a standard. Catalase (CAT) activity was measured in PMS by following the decomposition of the substrate H_2_O_2_ at 240 nm [35]. The conjugation of GSH with 1-chloro-2,4- dinitrobenzene (CDNB) at 340 nm was measured to determined glutathione-S-transferase (GST) activity in PMS [36]. The recycling reaction of reduced glutathione (GSH) with 5,5′-dithiobis-(2-nitrobenzoic acid) (DTNB) in the presence of glutathione reductase (GR) excess allowed to determine total glutathione (tGSH) content in the PMS fraction at 412 nm [37,38]. tGSH content was calculated as the rate of TNB^2-^ formation with an extinction coefficient of DTNB chromophore formed, ε = 14.1 × 103 M^−1^cm^−1^ [37,39]. Thiobarbituric acid-reactive substances (TBARS) were measured at 535 nm to determined endogenous lipid peroxidation (LPO) [40]. Protein carbonylation (PC) was measured at 450 nm based on the reaction of carbonyl groups with 2,4-dinitrophenylhydrazine (DNPH), according to the DNPH alkaline method [41]. LDH activity was measured by following the decrease of absorbance at 340 nm, as pyruvate consumption leads to NADH oxidation [42], adapted to microplate [16].

An ELISA assay to evaluate the HSP70/HSC70 content was performed as described in Vieira et al. [33]. The samples were added a primary antibody (1º Anti-HSP70 mouse mAB (C92F3A-5) Millipore), detecting 72 and 73 kDa proteins corresponding to the molecular mass of inducible hsp and hsc70, and a secondary antibody, anti-mouse IgC (2º Anti-mouse IgC (fab specific) Sigma), sequentially. The amount of HSP70/HSC70 was measured at 405 nm and a purified HSP70 active protein (HSP70 protein Millipore) used as a standard.

#### 2.4.3. Cellular Energy Allocation (CEA)

The content of sugars, lipids and proteins (summing the Ea—energy available) and the electron transport system (ETS) activity (estimation for Ec—aerobic energy production) were determined based on the methods of De Coen and Janssen [43] modified for microplate [44]. 

Muscle total lipid content was determined by adding chloroform, methanol, and ultra-pure water (2:2:1 proportion) to 300 μL of the homogenate. The organic phase of each sample, and tripalmitin as a standard, were and incubated with H_2_SO_4_ (15 min at 200 °C), and the absorbance measured at 375 nm. The fraction for carbohydrate content was obtained by adding 15% TCA to 300 μL of homogenate (incubated for 10 min at –20 °C). Carbohydrates quantification was performed in the supernatant by adding 5% phenol and H_2_SO_4_ to the samples, with glucose as a standard, and the absorbance read at 492 nm. The remaining pellet was used for total protein content quantification after resuspension with 1M NaOH (incubated for 30 min at 60 °C), and neutralization with 1.67 HCL. The Bradford’s method [34] was used for total protein content quantification using bovine serum albumin as a standard and absorbance measured at 520 nm. Lipids, sugars and proteins were converted into energetic equivalent values using the corresponding energy of combustion: 39,500, 17,500, 24,000 mJ/g [45].

Electron transport system (ETS) activity was read at 490 nm using the INT (Iodonitrotetrazolium) reduction assay, in which ETS is measured as the rate of INT reduction in the presence of the nonionic detergent Triton X-100. The stoichiometrical relationship in which for 2 μmol of formazan formed, 1 μmol of oxygen is consumed was used to calculate the cellular oxygen consumption rate. Ec value was obtained by the conversion to energetic values using the specific oxyenthalpic equivalent for an average lipid, protein and carbohydrate mixture of 480 kJ/mol O_2_ [45]. 

### 2.5. Statistical Analysis

Data normality was confirmed by using Kolmogorov-Smirnov test (*p* > 0.05). Brown-Forsythe test verified the homoscedasticity (*p* > 0.05). The parametric statistical two-way ANOVA was used to search for the significance of differences among *A. armata* exudate exposure, temperatures and their interactions. A pairwise multiple comparison procedure (Tukey test) was then used to test differences between treatments (control and *A. armata* exudate exposure) for both temperatures. GraphPad Prism version 6.00 for Windows (GraphPad Software, La Jolla, CA, USA) was used as software and statistically significant differences were considered when *p* < 0.05. Data were presented as mean value (mean) ± standard error value (SE). A full description of two-way ANOVAs results is presented in supplementary material.

## 3. Results and Discussion

The marine environment is constantly under pressure, and the expected warming associated with climate change may represent an additional impact to coastal organisms, modulating their fitness and survival. 

Both the halogenated compounds present in *A. armata* exudate and the temperature rise have the potential to induce ROS production that may reach levels above the acceptable [4,20], which may result in the activation or the impairment of antioxidant defenses in order to prevent oxidative damage. As a result, exposure to such stressful scenarios may also have metabolic and energetic consequences. Therefore, understand if the changes on oxidative and metabolic status of marine organisms imposed by the toxic exudate of the invasive seaweed *A. armata* are altered in a warming scenario is of primordial interest.

Overall, our results demonstrated that a short exposure to this predicted climate-change scenario may enhance some biochemical responses towards *A. armata* exudate exposure, providing a better understanding of the challenge such combination of stressors may pose to *M. galloprovincialis* individuals. 

**Figure 1:**
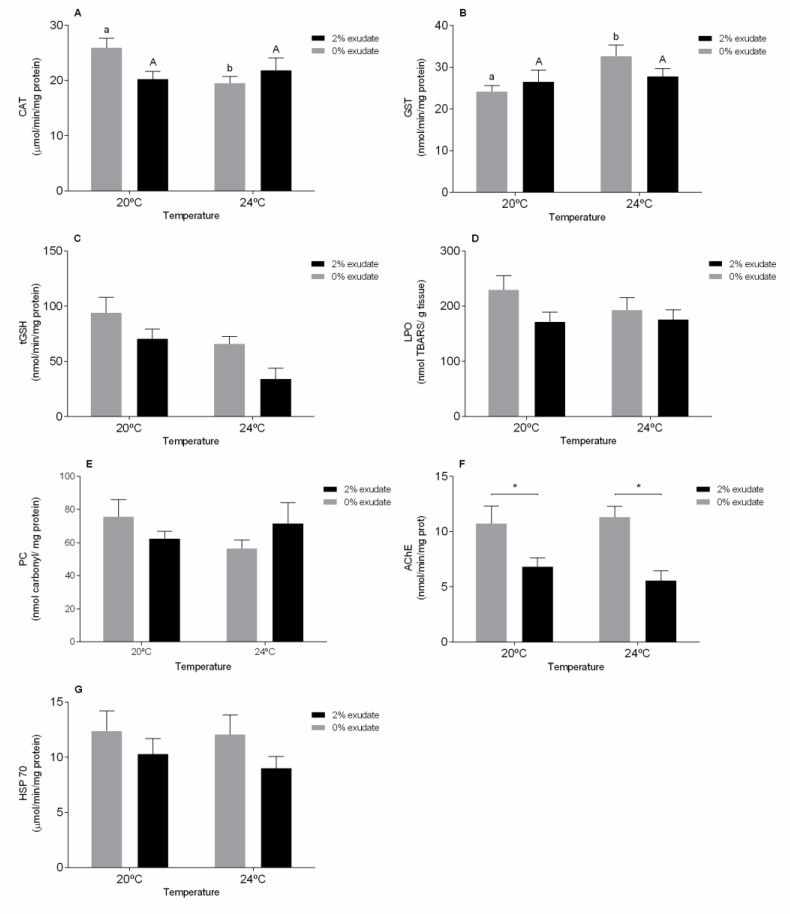
Biochemical biomarker responses of *M. galloprovincialis* gills after 96 h of exposure to *A. armata* exudate at different tested temperatures. (**A**) Catalase (CAT), (**B**) glutathione-S-transferase (GST), (**C**) total glutathione (tGSH), (**D**) lipid peroxidation (LPO), (**E**) protein carbonylation, (**F**) acetylcholinesterase (AChE), and (**G)** heat shock proteins (HSP70). Asterisk (*) indicates a significant difference compared with the 0% and 2% of *A. armata* exudate from the same temperature. Different lower-case letters point at differences in the 0% exudate treatments at the different temperatures and the upper-case letters indicate differences in the 2% exudate treatments.

The antioxidant defense system of the mussels involves enzymes such as CAT as first line of oxidative defense and GST and tGSH as second line. In the gills, significant effects were observed in the CAT activity as a result of the interaction of *A. armata* exudate exposure with the temperature (supplementary materials Table S1). Interestingly, a significant decrease (*p* < 0.05) in CAT activity was identified in the treatment without exudate from 20 °C to 24 °C (Figure 1A). The expected increased ROS production (especially the superoxide anion) favors the formation of H_2_O_2_ in the cells, since SOD activity results in high dismutation of O_2_^−^ to H_2_O_2_ [46]. Subsequently, this H_2_O_2_ can be converted into H_2_O and O_2_ by the action of CAT, thus preventing cell damage caused by oxidative stress [46]. Thus, the lower CAT activity observed in the treatment 0% exudate at 24 °C may be related to a strong involvement of CAT in the decomposition of H_2_O_2_. Our results are in line with Morosetti et al. [47], who also observed a significant inhibition of CAT activity in *M. galloprovincialis* in the control treatments with increasing temperature. Moreover, results suggest that following depletion of CAT activity, a significant induction of the GST activity as second line of defense occurred (Supplementary Materials Table S1) in the gills of individuals that were not exposed to the exudate (Figure 1B). Thus, these results point to the existence of a response towards oxidative stress derived from heat stress and are in line with other studies with different bivalve species in which an increase in the GST activity was also observed in similar conditions [32,48]. As CAT and GST, also tGSH plays a determinant role in the protection against ROS [48]. In addition to directly neutralizing several ROS through their oxidation in GSSG, tGSH also acts as a cofactor for several antioxidant enzymes [46]. Our results demonstrated significant changes in the levels of tGSH in the gills (Supplementary Materials Table S1) due to temperature and exposure to *A. armata* exudate. This decrease in tGSH levels observed mainly at 24 °C is consistent with the expected higher production of ROS in organisms exposed to warmer waters [4] and to the secondary metabolites present in seaweed exudate [49], suggesting that the decrease in tGSH levels observed in organisms exposed to *A. armata* exudate, especially at 24 °C, can be related with an active participation of tGSH in combating the ROS in excess (Figure 1C).

As a result, none of the experimental treatments resulted in oxidative damage in mussels’ gills (*p* > 0.05), measured as LPO and PC (Supplementary Materials Table S1, Figure 1D,E), revealing that antioxidant defenses in this tissue were effective under the tested conditions. In fact, our study corroborates the notion that gills tend to have a more efficient antioxidant machinery than other tissues as they are in direct contact with the external environment [32]. 

Concerning AChE activity in the gills, a noticeable effect was observed under exudate exposure (Supplementary Materials Table S1). Specifically, a significant decline (*p* < 0.05) was found between 0% and 2% *A. armata* exudate exposure treatments in both temperature scenarios (Figure 1F). AChE is an essential enzyme in the neurotransmission process [50], and the inhibition of AChE activity may result in neurological disorders that impair the behavior and ultimately culminate in the death of the exposed organisms [51]. This parameter has been often used to assess the neurotoxic impact of several compounds on marine organisms, being widely used as a biomarker of exposure to several biotic and abiotic stressors such as temperature and salinity [52] and algal toxins [53] and also environmental contaminants namely pesticides [54] and metals [55,56]. Some volatile halogenated organic compounds produced by *Asparagopsis* algae, such as haloacetones, are known enzyme inhibitors [13,14].The presence of these compounds may explain the inhibition of AChE activity observed in the gills of the exposed mussels, indicating potential neurotoxic effects of the exudate.

On the other hand, when considering the levels of stress protein HSP70 in the gills, no significant effects (*p* > 0.05) of *A. armata* exudate exposure, temperature and their interaction were observed (Supplementary Materials Table S1, Figure 1G). HSPs are typically expressed in response to thermal stress, playing a fundamental role in the folding of *de novo* synthesized proteins, as well as in the repair, refolding and degradation of damaged or denatured proteins [57].

**Figure 2:**
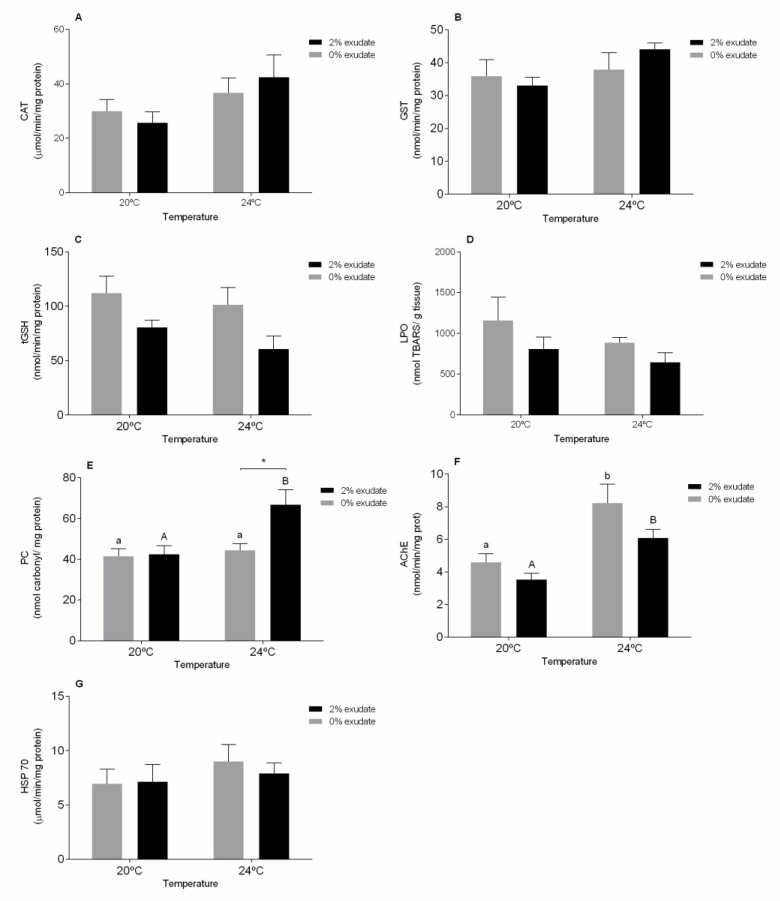
Biochemical biomarker responses of *M. galloprovincialis* digestive gland after 96 h of exposure to *A. armata* exudate at different temperatures. (**A**) Catalase (CAT), (**B**) glutathione-S-transferase (GST), (**C**) total glutathione (tGSH), (**D**) lipid peroxidation (LPO), (**E**) protein carbonylation, (**F**) acetylcholinesterase (AChE), and (**G)** heat shock proteins (HSP70). Asterisk (*) indicates a significant difference compared with the 0% and 2% of *A. armata* exudate from the same temperature and the different lower-case letters point at differences in the 0% exudate treatments at the different temperatures and the upper-case letters indicate differences in the 2% exudate treatments.

In the digestive gland, contrarily to what was observed in the gills, combined exposure to the exudate from *A. armata* and temperature did not alter CAT activity. On the other hand, a significant effect of temperature was demonstrated (*p* < 0.05, Supplementary Materials Table S2) although *post-hoc* tests did not detect statistical differences among treatments (Figure 2A). An increase in CAT activity may suggest that these organisms increased their antioxidants defenses, in an attempt to protect them from potential oxidative damage.

Considering the GST activity in the digestive gland, no significant effects (*p* > 0.05) of *A. armata* exudate exposure, temperature and their interaction were observed (Supplementary Materials Table S2). Attig et al. [58] did not observe changes on GST activity in digestive gland of *M. galloprovincialis* in the response to heat stress. When looking at tGSH levels in the digestive gland, these were significantly altered after exposure to *A. armata* exudate (*p* < 0.05, Supplementary Materials Table S2). As seen in the gills, tGSH levels in the digestive gland also decrease after exposure to the exudate (Figure 2C). Depletion of tGSH may be related to ROS detoxification, through the action of non-enzymatic antioxidant mechanisms. However, the decrease in tGSH levels can minimize the capacity to neutralize the ROS thus increasing the risk of damage by oxidative stress, which means that ROS induction by the secondary metabolites present in *A. armata* exudates may compromise the redox equilibrium of cells [59]. Similarly to the gills, LPO levels did not exhibit alterations in the digestive gland in none of the treatments (*p* > 0.05, Supplementary Materials Table S2, Figure 2D). On the other hand, significant oxidative damage at the proteins level (PC) was seen in the digestive gland of mussels exposed to the *A. armata* exudate, increased temperature and their combined exposure (Supplementary Materials Table S2). Mussels exposed to 2% *A. armata* exudate at 24 °C showed significantly higher PC values (*p* < 0.05) when compared to those of individuals exposed to 0% exudate at 24 °C or even than those exposed to 2% exudate at 20 °C (Figure 2E). PC results in the modification of amino acid aldehyde or ketone groups by ROS and can lead to inactivation or degradation of proteins’ functions [60]. Protein carbonylation (PC) is a form of oxidative damage, which may be promoted by enhanced production of reactive oxygen species [61], increasing its expression in response to different stressors, namely temperature [62], pesticides [60] and metals [33]. As both halocarbons and temperature rise are potent ROS generators [4,20], the inability to counteract ROS toxicity by the enzymatic and non-enzymatic antioxidant defences can result in cellular oxidative damages [46], as observed in our study.

Moreover, in the digestive gland, the response of AChE activity was significantly affected (*p* < 0.05) under increased temperature and also with exudate, revealing the neurotoxic potential of these stressors individually. A significant (*p* < 0.05) increase of AChE activity from 20 °C to 24 °C in both 0% and 2% treatments was observed (Figure 2F). However, when acting in combination these stressors did not alter this enzyme activity (Supplementary Materials Table S2). Enhanced AChE activity as a function of water temperature has been previously reported by Pfeifer et al. [52], who observed a rise in AChE activity during the summer months compared to the winter months in *Mytilus* sp. This AChE induction may be related to cell disrupting processes namely apoptosis [50]. Furthermore, although a slight increase was observed in the activity of HSP70 in the digestive gland in the same treatment, this was not significant (*p* > 0.05, Supplementary Materials Table S2).

**Figure 3:**
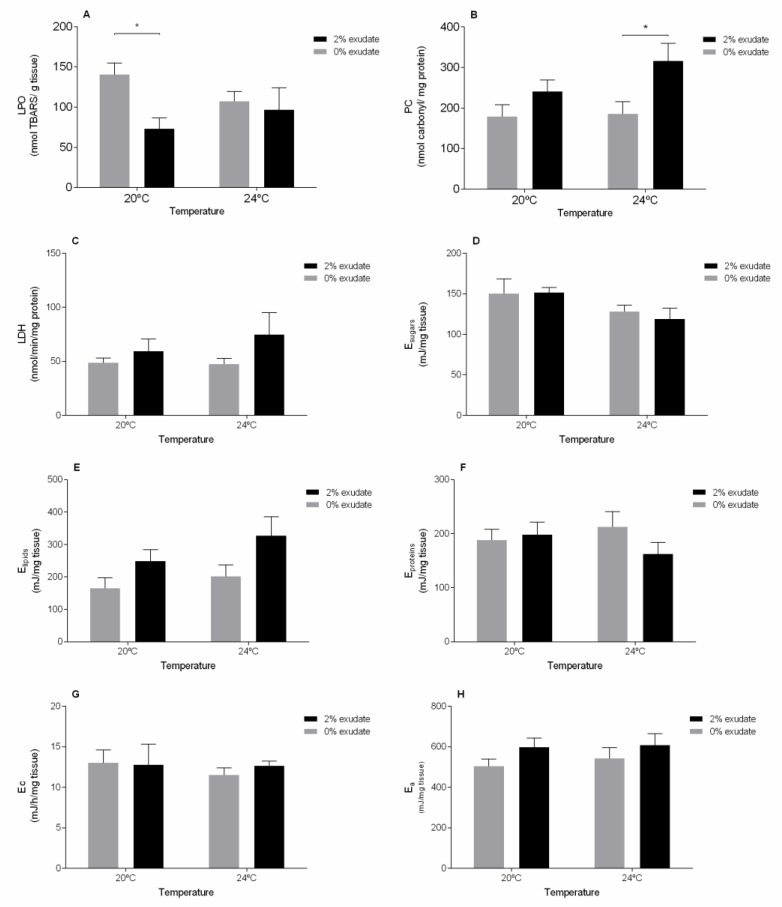
Biochemical biomarker responses of *M. galloprovincialis* muscle after 96 h of exposure to *A. armata* exudate at different temperatures. (**A**) Lipid peroxidation (LPO), (**B**) protein carbonylation (PC), (**C**) lactate dehydrogenase (LDH), (**D**) sugar content (E_sugar_), (**E**) lipid content (E_lipids_), (**F**) protein content (E_proteins_), (**G**) energy consumption (Ec), and (**H**) energy available. Asterisk (*) indicates a significant difference compared with the 0% and 2% of *A. armata* exudate from the same temperature.

Considering the oxidative damage in the muscle, and contrarily to the observed in the other tissues, seaweed exudate was the major trigger responsible for decreased LPO levels (*p* < 0.05, Supplementary Materials Table S3), specially at 20 °C (Figure 3A). This goes in line with the study by Coelho et al. [16] after exposing *M. galloprovincialis* to *A. armata* exudate at the same temperature; however, the noticed decrease was not significant. Furthermore, increased damage at the protein level (PC) was detected under warming (*p* < 0.05, Supplementary Materials Table S3). As observed for the digestive gland, PC levels in the muscle were significantly higher in individuals exposed to 2% exudate at 24 °C (Figure 3B), again suggesting the existence of damage by oxidative stress in the muscle tissue proteins in the presence of both stressors.

In relation to the LDH activity, no significant changes (*p* > 0.05) were detected due to the temperature, exposure to *A. armata* exudate or their co-occurrence (Supplementary Materials Table S3). Although not significant, LDH activity in the muscle of individuals exposed to 2% exudate was higher than those not exposed, at both temperatures (Figure 3C). LDH is an enzyme that plays an important role in the anaerobic pathway of energy production [63], especially when there is a sudden need for energy [64]. Alterations in LDH activity were found after exposure to different concentrations of cadmium [65], thiacloprid [66] and deltamethrin [67], demonstrating that changes in LDH activity can occur in situations of chemical stress. Thus, the tendency for an increase in anaerobic energy production may be related to a greater energy requirement of the organisms to combat the chemical stress caused by the exposure to the exuded halogenated compounds. At last, aerobic metabolic capacity (measured as ETS activity) of the mussels remained unaltered after being exposed to any of the experimental conditions (Figure 3G). Energy metabolism plays a crucial role in the survival of the organisms and their vital functions and potential to adapt to stressful conditions [57]. Conversely, a significant decrease in energy reserves in terms of sugar content (*p* < 0.05) was observed in relation to temperature but not between exudate treatments (*p* > 0.05), with only a slight decrease being observed between 20 °C and 24 °C (Figure 3D). This may reflect a higher energetic expenditure in mussels exposed to warming conditions, possibly due to increased investment on antioxidant defense mechanisms. A similar response was observed in *M. galloprovincialis* exposed to temperature rise under different tidal emersion cycles [27]. In contrast, exposure to *A. armata* exudate resulted in significant changes (*p* < 0.05) in the lipid content (Supplementary Materials Table S3). An increase in lipids was depicted in mussels exposed to the exudate at 20 °C and 24 °C (Figure 3E). Similarly, Coelho et al. [16] observed the same response in *M. galloprovincialis* exposed to exudate from *A. armata*. Lipids are essential constituents of biologically active molecules as well as important energy reserves in bivalves [16]. An increase in lipid content after exposure to chemicals may be related to behavioral changes (e.g., dietary and physiological), resulting in lower energy expenditure by the individuals (Verslycke et al. 2004). However, these alterations were not reflected in terms of protein content nor in the overall energy available (Ea) (Supplementary Materials Table S3, Figure 3G,H, respectively). 

## 4. Conclusions

In a context of climate change-related warming and the growing spread of biological invasions, this study provides relevant data on the potential risk of the presence of the invasive red seaweed *Asparagopsis armata* in coastal communities, namely in the mussel *Mytilus galloprovincialis*. 

Overall, exposure to *A. armata* exudate challenges antioxidant defenses in *M. galloprovincialis*, especially under a warming scenario. It is also highlighted that the degree of tissue specialization may lead to different responses and capability to counteract oxidative stress. The antioxidant response in the gills seems to be more effective in combating cellular oxidative damage. However, in other *M. galloprovincialis* tissues (digestive gland and muscle), oxidative damage at the protein level could not be prevented after being exposed to *A. armata* exudate under warmed seawater. In addition, exposure to *A. armata* exudate appears to alter some energy reserves without compromising metabolic capacity. The neurotoxic potential of the *A. armata* exudate was also determined, especially in the gills, in both temperature regimes.

Thus, this study identified some potential consequences of exposure to *A. armata* exudate under temperature rise, demonstrating the vulnerability of *M. galloprovincialis* to this invasive seaweed exposure in future climate change scenarios.

## Data Availability

The data presented in this study is available in the current manuscript, raw data is available on request from the corresponding author.

## Supplementary Materials

The following are available online at https://www.mdpi.com/article/10.3390/toxics9060121/s1, Table S1: results for two-way ANOVA analysis on biochemical biomarkers responses in the gills of *Mytilus galloprovincialis* with *A. armata* exudate exposure and water temperature as factors, Table S2: results for two-way ANOVA analysis on biochemical biomarkers responses in the digestive gland of *Mytilus galloprovincialis* with *A. armata* exudate exposure and water temperature as factors, Table S3: results for two-way ANOVA analysis on biochemical biomarkers responses in the muscle of Mytilus galloprovincialis with A. armata exudate exposure and water temperature as factors.
