# Supplementary material for: Ocean Warming May Enhance Biochemical Alterations Induced by an Invasive Seaweed Exudate in the Mussel Mytilus galloprovincialis
Source: Toxics. 2021 May 28;9(6):121. doi: 10.3390/toxics9060121 (PMC8229087; doi:10.3390/toxics9060121)
Supplement: Supplementary file 1 [file toxics-09-00121-s001.zip › toxics-1240947-supplementary.pdf]

toxics

Supplementary Materials: Ocean Warming May Enhance Biochemical Alterations Induced by an Invasive Seaweed Exudate in the Mussel *Mytilus galloprovincialis*

Hugo C. Vieira, Andreia C.M. Rodrigues, Sílvia F.S. Pires, Jacinta M.M. Oliveira, Rui J.M. Rocha, Amadeu M.V.M. Soares and Maria D. Bordalo

Table S1. Results for two-way ANOVA analysis on biochemical biomarkers responses in the gills of *Mytilus galloprovincialis* with *A. armata* exudate exposure and water temperature as factors.

Biomarker.	Factor	df	F	*p*	% of Total Variation
	Exudate	1, 24	0.95	0.33	2.94
CAT	Temperature	1, 24	2.02	0.16	6.22
	Interaction	1, 24	5.47	**0.03**	16.85
	Exudate	1, 24	0.28	0.60	0.88
GST	Temperature	1, 24	4.66	**0.04**	14.80
	Interaction	1, 24	2.55	0.12	8.10
	Exudate	1, 20	6.35	**0.02**	18.91
tGSH	Temperature	1, 20	8.67	**0.01**	25.8
	Interaction	1, 20	0.15	0.70	0.45
	Exudate	1, 24	4.45	0.08	14.11
LPO	Temperature	1, 24	0.27	0.27	4.05
	Interaction	1, 24	0.19	0.45	5.69
	Exudate	1, 24	0.01	0.91	0.04
PC	Temperature	1, 24	0.30	0.59	1.13
	Interaction	1, 24	2.54	0.12	9.45
	Exudate	1, 23	157	**< 0.01**	43.75
AChE	Temperature	1, 23	0.09	0.77	0.21
	Interaction	1, 23	0.68	0.42	1.63
	Exudate	1, 24	2.76	0.11	10.17
HSP70	Temperature	1, 24	0.27	0.61	0.98
	Interaction	1, 24	0.10	0.76	0.36

Table S2. Results for two-way ANOVA analysis on biochemical biomarkers responses in the digestive gland of *Mytilus galloprovincialis* with *A. armata* exudate exposure and water temperature as factors.

**Biomarker**	**Factor**	**df**	**F**	***p***	**% of Total Variation**
CAT	Exudate	1, 23	0.02	0.89	0.07
	Temperature	1, 23	4.59	**0.04**	16.24
	Interaction	1, 23	0.83	0.37	2.92
GST	Exudate	1, 24	0.83	0.37	2.69
	Temperature	1, 24	3.82	0.06	12.32
	Interaction	1, 24	2.33	0.14	7.53
tGSH	Exudate	1, 22	6.98	**0.01**	23.46
	Temperature	1, 22	1.26	0.27	4.25
	Interaction	1, 22	0.11	0.74	0.38
LPO	Exudate	1, 24	2.80	0.11	9.85
	Temperature	1, 24	1.55	0.23	5.45
	Interaction	1, 24	0.10	0.75	0.36
PC	Exudate	1, 24	5.55	**0.03**	13.32
	Temperature	1, 24	7.51	**0.01**	18.02
	Interaction	1, 24	4.61	**0.04**	11.06
AChE	Exudate	1, 23	4.99	**0.03**	10.36
	Temperature	1, 23	18.62	**< 0.01**	38.64
	Interaction	1, 23	0.58	0.45	1.20
HSP70	Exudate	1, 24	0.11	0.74	0.44
	Temperature	1, 24	1.02	0.32	4.01
	Interaction	1, 24	0.22	0.64	0.88

Table S3. Results for two-way ANOVA analysis on biochemical biomarkers responses in the muscle of *Mytilus galloprovincialis* with *A. armata* exudate exposure and water temperature as factors.

Biomarker	Factor	df	F	*p*	% of Total Variation
LPO	Exudate	1, 24	5.96	**0.02**	17.47
	Temperature	1, 24	0.51	0.48	1.49
	Interaction	1, 24	3.64	0.07	10.68
PC	Exudate	1, 24	8.38	**0.01**	23.93
	Temperature	1, 24	1.54	0.23	4.39
	Interaction	1, 24	1.08	0.31	3.10
LDH	Exudate	1, 24	2.39	0.14	8.78
	Temperature	1, 24	0.33	0.57	1.23
	Interaction	1, 24	0.47	0.50	1.73
Sugars	Exudate	1, 24	0.11	0.74	0.38
	Temperature	1, 24	4.99	**0.03**	17.06
	Interaction	1, 24	0.17	0.68	0.59
Lipids	Exudate	1, 24	6.30	**0.02**	19.41
	Temperature	1, 24	1.90	0.18	5.87
	Interaction	1, 24	0.25	0.62	0.76
Proteins	Exudate	1, 23	0.30	0.59	1.25
	Temperature	1, 23	0.01	0.94	0.02
	Interaction	1, 23	0.95	0.34	3.91
Ec	Exudate	1, 23	0.07	0.79	0.29
	Temperature	1, 23	0.24	0.63	1.01
	Interaction	1, 23	0.17	0.68	0.72
Ea	Exudate	1, 24	2.76	0.11	10.17
	Temperature	1, 24	0.62	0.62	0.93
	Interaction	1, 24	0.77	0.77	0.32
